# CCN5 attenuates profibrotic phenotypes of fibroblasts through the Smad6-CCN2 pathway: Potential role in epidural fibrosis

**DOI:** 10.3892/ijmm.2015.2190

**Published:** 2015-04-21

**Authors:** HONGHAI XU, CONG LIU, ZHENGMING SUN, XIONG GUO, YUELIN ZHANG, MENGTING LIU, PENG LI

**Affiliations:** 1 Department of Orthopaedics, The Third Affiliated Hospital (Shaanxi Provincial People’s Hospital), Medical College of Xi’an Jiaotong University, Xi’an, Shaanxi 710061, P.R. China; 2 Xi’an Medical College of Xi’an Jiaotong University, Xi’an, Shaanxi 710061, P.R. China; 3 Department of Public Health, Medical College of Xi’an Jiaotong University, Xi’an, Shaanxi 710061, P.R. China; 4 Department of Neurosurgery, The Third Affiliated Hospital (Shaanxi Provincial People’s Hospital), Medical College of Xi’an Jiaotong University, Xi’an, Shaanxi 710061, P.R. China

**Keywords:** epidural fibrosis, CCN5, CCN2, Smad6, cell proliferation, fibrotic phenotype

## Abstract

Epidural fibrosis is characterized by the development of dense and thick scar tissue adjacent to the dural mater and ranked as the major contributor for post-operative pain recurrence after laminectomy or discectomy. Recently, CCN5 exhibited an inhibitory effect on connective tissue growth factor (CTGF)/CCN2 (a critical regulator for fibrotic disease)-mediated fibrogenesis. However, its function in epidural fibrosis and the underlying mechanisms involved remain to be determined. In this study, an obvious downregulation of CCN5 was observed in scar tissues from laminectomized rats, concomitant with a marked upregulation of CCN2, suggesting a potential negative regulatory role of CCN5 in fibrogenesis. Furthermore, CCN5 overexpression notably mitigated transforming growth factor-β1-enhanced fibroblast viability and proliferation. Of note, CCN5 upregulation inhibited the switch of fibroblasts into myofibroblasts as its overexpression abrogated the expression of the myofibroblast marker, α-smooth muscle actin (α-SMA). CCN5 upregulation also reduced an increase in collagen type I, α1 (COL1A1) and total collagen concentrations. Additionally, CCN5 over expression decreased CCN2 expression and increased Smad6 phosphorylation. Mechanism analysis revealed that blocking Smad6 signaling significantly ameliorated the inhibitory effect of CCN5 on the CCN2 levels, accompanied by the reduction in cell proliferation and collagen production. These results confirm that CCN5 exerts an anti-fibrotic function by regulating the Smad6-CCN2 pathway, thereby indicating a potential approach for ameliorating epidural fibrosis after laminectomy.

## Introduction

Epidural fibrosis (EF) ranks as a common complication of laminectomy, a common type of surgery utilized to treat spinal diseases, including lumbar disc herniation, lumbar (low back) spinal stenosis and other lumbar disorders (1,2). Extensive scar adhesion between the dural mater and surrounding muscles may contribute to the formation of EF and subsequently cause post-operative pain recurrence after laminectomy or discectomy. Large therapeutic schedules are utilized to prevent epidural scar adhesion. Accordingly, the reduction of scar-triggered epidural fibrosis is crucial for the treatment of spinal diseases.

Scar formation and adhesion often lead to negative effects on outcome after laminectomy. Previous findings have demonstrated that fibroblast hyperplasia is critical for epidural fibrosis as it plays important roles in the generation of the fibrotic matrix during scar formation (3,4). As a highly mechano sensitive cell-type, fibroblasts transform into profibrotic myofibroblasts to enhance the expression of α-smooth muscle actin (α-SMA) and extracellular matrix (ECM) proteins, which play a vital role in wound repair and scar formation (5,6). Accumulating evidence has indicated that blocking fibroblast proliferation with 10-hydroxycamptothecin (HCPT) significantly reduces the degree of epidural adhesion after laminectomy, indicating a potential strategy for the improvement of the surgical curative effect (3).

The connective tissue growth factor/cysteine-rich 61/nephroblastoma overexpressed (CCN) family comprises six highly conserved cysteine rich proteins. Mounting evidence suggests the critical roles of CCN family in various cell processes including proliferation and adhesion, homeostasis, osteoblast differentiation, wound repair, inflammation, fibrosis and tumorigenesis (7–9). Of these members, connective tissue growth factor (CTGF)/CCN2 acts as an ECM protein and is associated with wound healing and scar formation (10,11). The downregulation of CCN2 expression with its specific anti-sense oligonucleotides significantly decreases myofibroblast numbers, collagen formation and limits scar hypertrophy (10).

CCN5 (also known as rCop-1, Wisp-2 and CTGF-l) is another key member of the CCN5 family and is located on chromosome 20q12-q13.1. Unlike other members, CCN5 is absent in the cysteine knot (CT) containing a carboxyl-terminal domain. Previous studies have demonstrated a growth inhibition effect of CCN5 on smooth muscle cell proliferation and motility (12). Furthermore, an inhibitory effect of CCN5 on CCN2-mediated fibrogenesis has been identified, suggesting a novel anti-fibrotic function of CCN5 (13). CCN2 and CCN5 were found to be upregulated during the development of cardiac hypertrophy. However, CCN2 expression accelerates cardiac fibrosis and hypertrophy, whereas CCN5 exerted anti-hypertrophic and -fibrotic effects, indicating that CCN5 antagonizes CCN2 function during the development of cardiac hypertrophy and fibrosis (14). Moreover, a negative regulatory function of CCN5 on α-SMA and collagen I expression was confirmed (15). Additionally, CCN5 repressed transforming growth factor-β (TGF-β) signaling pathway, which plays a pivotal role in scar adhesion and epidural fibrosis after laminectomy (16). However, CCN5 function in epidural fibrosis and its underlying mechanism remain unclear.

In the present study, the expression levels of CCN5 analyzed in laminectomized rats. The isolated fibroblasts were employed in order to explore the proliferative and fibrotic effect of CCN5. Furthermore, the underlying mechanisms were investigated.

## Materials and methods

### Reagents

Unless stated otherwise, all the substances were purchased from Gibco (Grand Island, NY, USA). The primary antibodies against CCN2 (ab6992) were purchased from Abcam (Cambridge, MA, USA). Anti-CCN5 antibodies (sc-12010) were purchased from Santa Cruz Biotechnology, Inc. (Santa Cruz, CA, USA). Anti-phospho-Smad6 and Smad6 antibodies (9519) were purchased from Cell Signaling Technology (Beverly, MA, USA). Rabbit anti-α-SMA polyclonal antibody (ab5694) was purchased from Abcam (Cambridge, UK). Rabbit polyclonal antibody raised against COL1A1 was obtained from Abnova Corp. (Taipei, Taiwan).

### Animal models

Twenty-four healthy 12-week-old male Lewis rats were provided by the Laboratory Animal Center of Xi’an Jiaotong University and included in this study. The rat laminectomy models were performed as previously described (4). Animal experiments were undertaken follwing the approval of the Institutional Animal Care and Use Committee. Briefly, the rats were anaesthetized with sodium pentobarbital (50 mg/kg). Prior to laminectomy, all the animals were shaved in the area near the first lumbar vertebra (L1) and the third lumbar vertebra (L2). The exposed skin was sterilized, followed by a midline skin incision. The dura mater of the L1 vertebrae was exposed after removing the spinous processes. A total laminectomy at L2 vertebra was performed by a rongeur. Fourteen days later, the rats were sacrificed, the scar and the surrounding normal tissues were collected for subsequent analysis.

### Fibroblast isolation and culture in vitro

The primary fibroblasts were obtained from the tail skin of rats. The protocol conformed to the guidelines of the Institutional Animal Care and Use Committee. Following enzymatic digestion, the isolated cells were incubated in Dulbecco’s modified essential medium (DMEM) (Life Technologies, Gaithersburg, MD, USA) containing 10% fetal bovine serum (FBS) (Invitrogen, Grand Island, NY, USA), 100 *µ*g/ml streptomycin and penicillin. The cells were stimulated with TGF-β1 (10 ng/ml) (Sigma, St. Louis, MO, USA) for 12 h and cultured in a humidified atmosphere at 37°C with 5% CO_2_. Fibroblasts from passages 4 to 6 were used in the subsequent study.

### Lentivirus construction and infection

To construct the recombinant lentiviral vector carrying CCN5, the full-length CCN5 cDNA was amplified with its specific primers (sense, 5′-GCCGCGTGGGACACGGTGACATGAGG-3′, containing *Mlu*I restriction enzyme site; 5′-CGGTCGACCAGTTGGCCTTAGAAAGC-3′, containing *Sal*I restriction enzyme site). Following digestion with *Mlu*I and *Sal*I restriction enzymes, the CCN5 cDNA was ligated into the lentivirus plasmid pWPT-GFP (Toyobo, Tokyo, Japan) to construct the recombinant pWPT-CCN5 plasmids, which contained the green fluorescent protein (GFP) and were digested with *Mlu*I and *Sal*I restriction enzymes. The 293 cells were then co-transfected with the lentivirus plasmids pWPT-CCN5, packaging vectors of pCMV-VSV-G and pCMV-dR8.91 (Clontech, Saint-Germain-en-Laye, France) using Lipofectamine^®^ 2000 Reagent (Invitrogen-Life Technologies, Carlsbad, CA, USA) at 37°C, 5% CO_2_ for 12 h. The cultured medium was collected and filtered. The cultured fibroblasts were then infected with the collected LV-CCN5 adenovirus. The virus was amplified and purified. Virus titers were determined by p24 ELISA kit (Cell Biolabs, Inc., San Diego, CA, USA), and then stored at −80°C for use. The vectors were used as a negative control.

### Transfection with Smad6 siRNA

To silence Smad6 levels in fibroblasts, the specific siRNA fragments of Smad6 and control siRNA were designed as previously described (17) and synthesized by Takara. For transfection, the cells were seeded into 6-well plates and grown to 40-50% confluence. Subsequently, 2 *µ*g/ml Smad6 siRNA or control siRNA was transfected into the cells using Lipofectamine 2000 (Invitrogen-Life Technologies) according to the manufacturer’s instructions. The transfection efficiency was analyzed by western blotting. Data are reported as the mean of three or four distinct experiments.

### RNA extraction and real-time polymerase chain reaction (PCR)

Total RNA was extracted using TRIzol reagent according to the manufacturer’s instructions (Biostar, Shanghai, China). Approximately 5 *µ*g of RNA was reverse transcribed to synthesize the first-strand cDNA using the cDNA Synthesis kit (Fermentas, St. Leon-Rot, Germany). Real-time PCR was performed at a final volume of 20 *µ*l which consisted of 10 *µ*l SYBR^®^ Premix Ex Taq™ II, 10 *µ*mol/l specific primers, 4 *µ*l of DNA, and H_2_O. The specific primers used were: CCN2, 5′-TAG CAAGAGCTGGGTGTGTG-3′ (sense) and 5′-TTCACTTGC CACAAGCTGTC-3′ (antisense); CCN5, 5′-TTAGCACTTGTG GTGGCTTG-3′ (sense) and 5′-CCATTGAGAGAAGGCAG AGG-3′ (antisense); collagen type I, α1 (COL1A1), 5′-ATCAGCCCAAACCCCAAGGAGA-3′ (sense) and 5′-CGCAGGAAG GTCAGCTGGATAG-3′ (antisense). The above mRNA levels were normalized to β-actin. All the samples were performed in triplicate.

### Western blot analysis

After lysis with RIPA lysis buffer (100 mM NaCl, 50 mM Tris-HCl pH 7.5, 1% Triton X-100, 1 mM EDTA, 10 mM β-glycerophosphate, 2 mM sodium vanadate and protease inhibitor), total protein extracts were analyzed by the BCA protein assay (Pierce, Rockford, IL, USA). Then, 200 *µ*g protein was separated by SDS-PAGE and transferred to a PVDF membrane. After blocking with 5% non-fat milk, the membrane was incubated with the primary antibodies against CCN2, CCN5, Smad-6, p-Smad6, α-SMA and COL1A1, followed by incubation with the corresponding secondary antibodies to horseradish peroxidase (HRP). The proteins were detected with enhanced chemiluminescence (ECL; Amersham Pharmacia Biotech, Piscataway, NJ, USA) and normalized with β-actin.

### [^3^H]-Tzhymidine incorporation assay ([^3^H]-TdR)

The cells were seeded in 24-well plates with the density of 4×10^4^ cells/well. The cells were cultured in DMEM medium for 24 h, followed by the serum-starved incubation for 2 h with the indicated treatments. After treatment with [^3^H]-thymidine (Sigma) for 6 h, the cells were washed three times with ice-cold normal saline. Trichloroacetic acid (10%) was added for a further 30-min incubation at 4°C. The liquid scintillation counter (Beckman Coulter, Fullerton, CA, USA) was introduced to evaluate cell proliferation by detecting the radioactivity.

### MTT assays

The MTT assay was performed to evaluate cell viability. Briefly, the cells were seeded in 24-well plates with a density of 1×10^5^ cells/well. After preconditioning with the above indicated treatments, 20 *µ*l MTT reagent (Sigma) was added for 6 h at 37°C, followed by treatment with 200 *µ*l isopropanol to dissolve formazan production. Cell viability was then evaluated by analyzing the absorbance of MTT at 590 nm using a micro-ELISA reader (Bio-Rad, Hercules, CA, USA). The samples were performed in triplicate and the results were presented as the percentage of growth inhibition.

### Measurement of collagen protein

Following treatment as described above, the total soluble collagen in culture supernatants was detected using a Sircol Assay kit (Biocolor, Belfast, Northern Ireland, UK), according to the manufacturer’s instructions.

### Statistical analysis

Data are shown as the means ± SD from at least three experiments. The Student’s t-test was used to assess the statistical significance differences in multiple comparisons. Data were analyzed using SPSS 11.0 software. P<0.05 was considered to indicate a statistically significant difference.

## Results

### Expression levels of CCN5 and CCN2 in scar tissue after laminectomy

**Figure 1:**
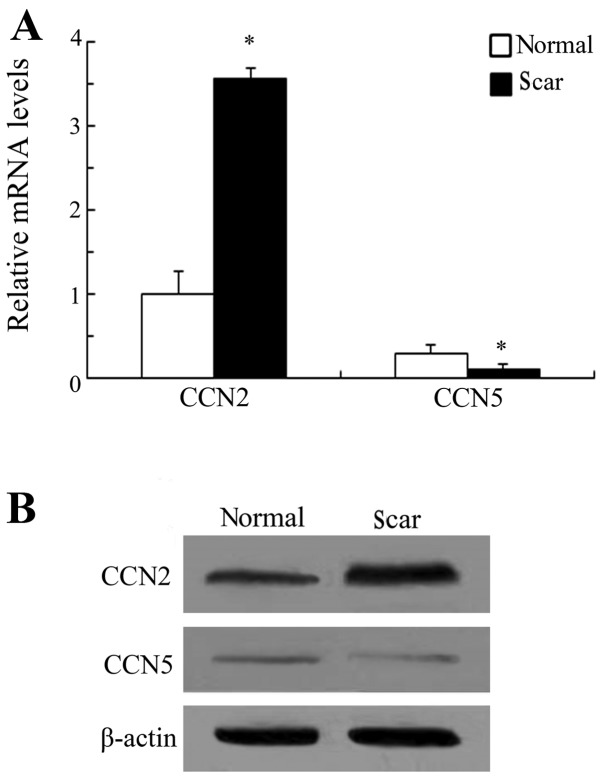
Expression of CCN5 and CCN2 in scar tissues after laminectomy. To determine the expression levels of CCN5 and CCN2, 24 healthy male Lewis rats (12 weeks old) were subjected to laminectomy. Then, the scar and surrounding normal tissues were collected. The expression levels of CCN5 and CCN2 were detected by RT-PCR (A). The corresponding protein levels were also evaluated by western blotting (B). ^*^P<0.05 vs. normal tissues.

CCN5 exerted an opposing function in CCN2-induced cardiac hypertrophy and fibrosis (14). However, its roles in scar-triggered epidural fibrosis remain to be determined. Laminectomized rats were used to determine the expression levels of CCN5 and CCN2 in scar tissue after laminectomy. RT-PCR analysis confirmed an obvious upregulation of CCN2 in scar tissues as compared to the surrounding normal tissues (Fig. 1A). A similar upregulation in CCN2 protein levels was demonstrated by western blotting (Fig. 1B). However, CCN5 expression levels were significantly downregulated during scar formation following laminectomy. CCN2 has been believed to act as a positive regulator of fibrogenesis, scar formation and wound repair (11). Therefore, these results suggest that CCN5 elicits potential anti-fibrotic effects during the development of scar formation based on opposing expression levels to CCN2 in scar tissues.

### rCCN5 transfection enhances CCN5 expression in primary fibroblasts

**Figure 2:**
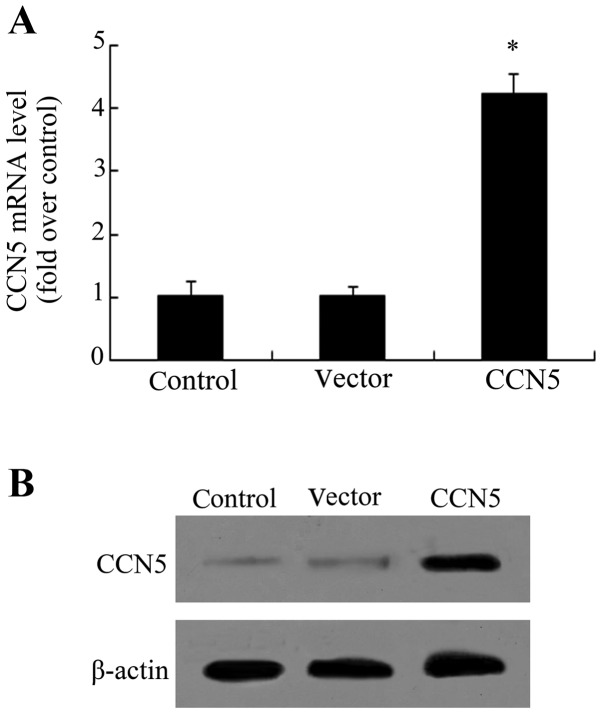
CCN5 transfection enhances CCN5 expression in primary fibroblasts. Primary fibroblasts were obtained from the tail skin of rats and cultured in DMEM medium. Lentivirus plasmid pWPT-GFP was introduced to construct the recombinant pWPT-CCN5 plasmids, following packaging with vectors of pCMV-VSV-G and pCMV-dR8.91. The corresponding transfection effect of CCN5 mRNA (A) and protein levels (B) was assessed individually with RT-PCR and western blotting. ^*^P<0.05.

Fibroblasts are essential for epidural fibrosis due to their function in fibrotic matrix formation during scar formation (3,4). To investigate the effect of CCN5 on scar formation, the recombinant lentiviral vector-carrying CCN5 (LV-CCN5) was constructed and transfected into the isolated primary fibroblasts. As shown in Fig. 2A, an ~4.1-fold increase in CCN5 mRNA levels was observed when the cells were transfected with LV-CCN5. Furthermore, western blot analysis confirmed the obvious upregulation of CCN5 protein following LV-CCN5 transfection, compared with the control and vector-treated groups (Fig. 2B), indicating that a stable overexpression system of CCN5 had been successfully constructed.

### CCN5 overexpression inhibits cell viability and proliferation

**Figure 3:**
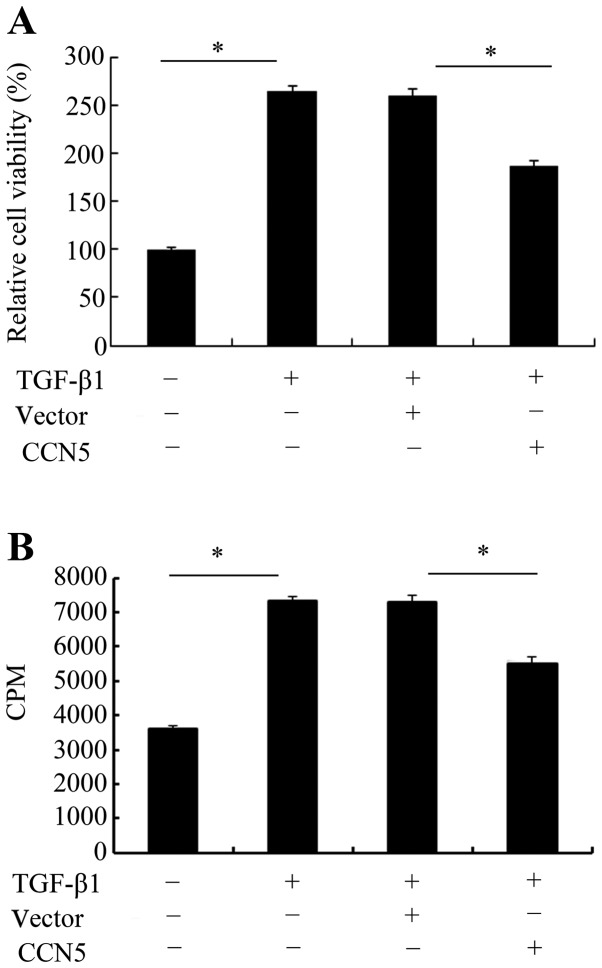
The function of CCN5 on fibroblast cell proliferation and viability. To assess the effect of CCN5 on cell proliferation and viability in response to the transforming growth factor-β (TGF-β) (10 ng/ml) for 12 h, cells were seeded in 24-well plates and treated with LV-CCN5, or vector transfection. Then, 20 *µ*l MTT reagent was added for 6 h to determine the roles of CCN5 overexpression in cell viability (A). Furthermore, the corresponding effects of CCN5 on cell proliferation were analyzed by addition of [^3^H]-thymidine (B). ^*^P<0.05.

TGF-β1 is known to be critical for scar-triggered epidural fibrosis following laminectomy due to its important role in the development of fibrosis (16). Based on the stable expression of CCN5 in fibroblasts, cell viability and proliferation in response to TGF-β1 was investigated. An MTT assay was used to assess the effect of CCN5 on cell viability. As shown in Fig. 3A, TGF-β1 stimulation exhibited a 2.5-fold increase in cell viability. However, this increase was markedly attenuated when CCN5 was overexpressed in fibroblasts. Further [^3^H]-TdR analysis confirmed that TGF-β1 treatment induced cell proliferation and resulted in an ~2.0-fold increase in cell numbers (Fig. 3B). Elevated CCN5 expression decreased TGF-β1-induced cell proliferation. These results suggested that CCN5 exhibits a negative inhibitory function during the TGF-β1-induced process of fibrosis by inhibiting fibroblast cell viability and proliferation.

### Elevated CCN5 expression reduces the profibrotic phenotype of fibroblasts induced by TGF-β1

**Figure 4:**
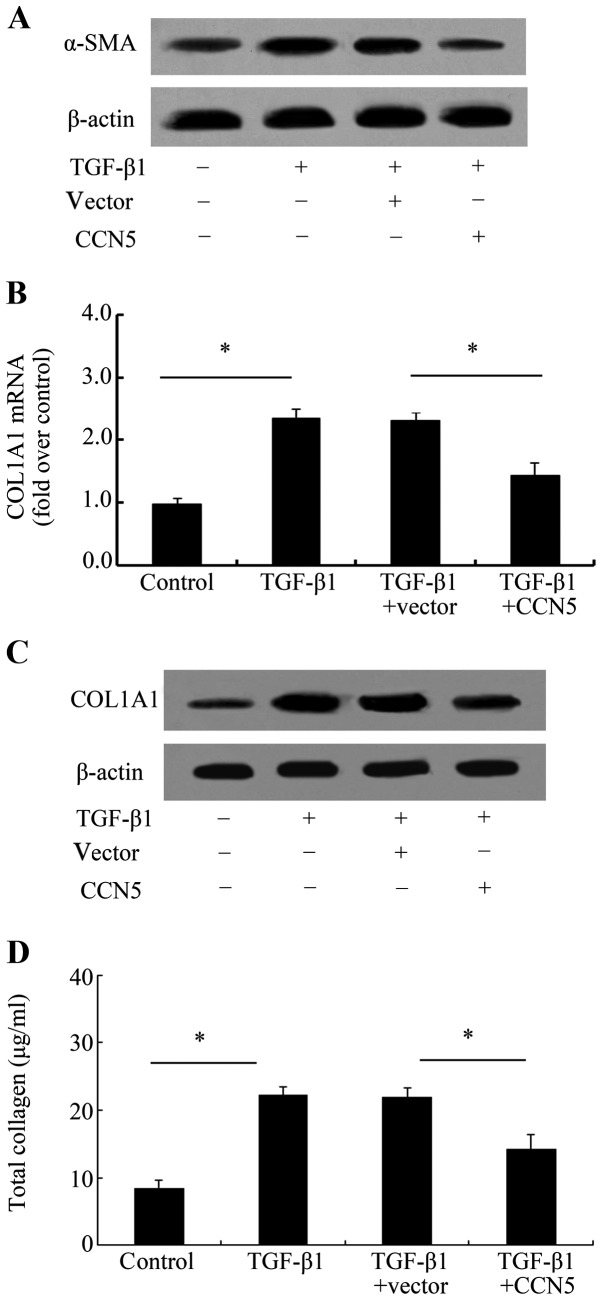
Effect of CCN5 on the profibrotic phenotype of fibroblasts induced by transforming growth factor-β (TGF-β1). The isolated fibroblasts were transfected or not with LV-CCN5 prior to TGF-β1 stimulation. The expression levels of α-smooth muscle actin (α-SMA), a specific marker of myofibroblasts, were analyzed by western blotting (A). mRNA (B) and protein levels (C) of CLO1A1 were asssessed. The total collagen concentration was assessed using a Sircol assay kit (D). ^*^P<0.05.

To assess the function of CCN5 during the development of scar formation, the anti-fibrotic effect of CCN5 was examined. α-SMA is believed to be a biochemical marker of myofibroblasts transformed from fibroblasts, which result in the excessive accumulation of fibrotic tissue and subsequent scar formation and adhesion (18). Therefore, we assessed the effect of CCN5 on α-SMA and the results confirmed that CCN5 overexpression markedly decreased α-SMA levels induced by TGF-β1 (Fig. 4A), suggesting that CCN5 attenuated the transformation of fibroblasts into profibrotic myofibroblasts. COL1A1 is a major ECM protein that is able to induce collagen I formation, which is the most abundant product of fibrosis. Further analysis exhibited a notable upregulation in COL1A1 mRNA levels (Fig. 4B) and protein levels (Fig. 4C) after TGF-β1 stimulation. However, this increase was obviously attenuated when preconditioning with LV-CCN5 transfection. Simultaneously, CCN5 over expression also markedly downregulated the total collagen concentration (Fig. 4D), indicating that CCN5 antagonized TGF-β1-induced profibrotic phenotype of fibroblasts.

### CCN5 attenuates TGF-β1-induced CCN2 expression

**Figure 5:**
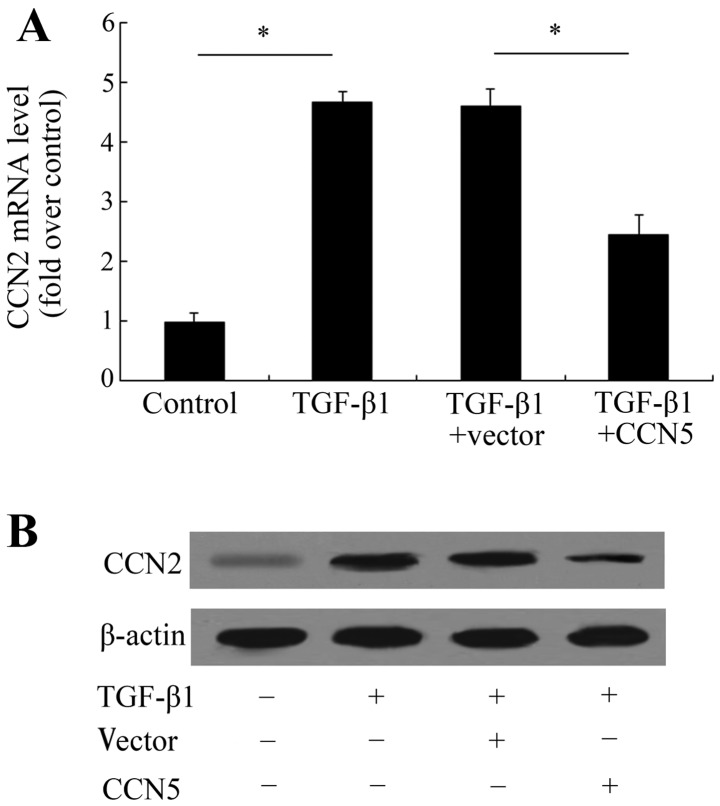
CCN5 overexpression attenuates CCN2 levels. Following transfection with the recombinant CCN5, the cells were stimulated with transforming growth factor-β (TGF-β1). The expression levels of CCN2 mRNA (A) and protein (B) were detected by RT-PCR and western blotting, respectively. ^*^P<0.05.

CCN2 is overexpressed in tissue repair and human diseases characterized by excessive scarring and fibrosis allowing it to be induced by TGF-β1 thereby enhancing the progressive fibrotic response to scar tissue formation (11,19). Based on the opposing expression levels of CCN5 and CCN2 in scar tissues, we analyzed the correlation between CCN5 and CCN2 during the fibrotic process of fibroblasts. TGF-β1 treatment induced an obvious upregulation of CCN2 mRNA levels (Fig. 5A). Further assay showed that CCN5 expression significantly attenuated this increase in CCN2 mRNA from 4.59- to 2.45-fold. The protein levels of CCN2 were also decreased following LV-CCN5 treatment, compared with the TGF-β1-treated group (Fig. 5B). These data indicate that CCN5 may exert anti-fibrotic effects by inhibiting the activation of the CCN2 pathway.

### CCN5 functions in TGF-β1-induced proliferation and profibrotic phenotype through Smad6-CCN2 pathway

**Figure 6:**
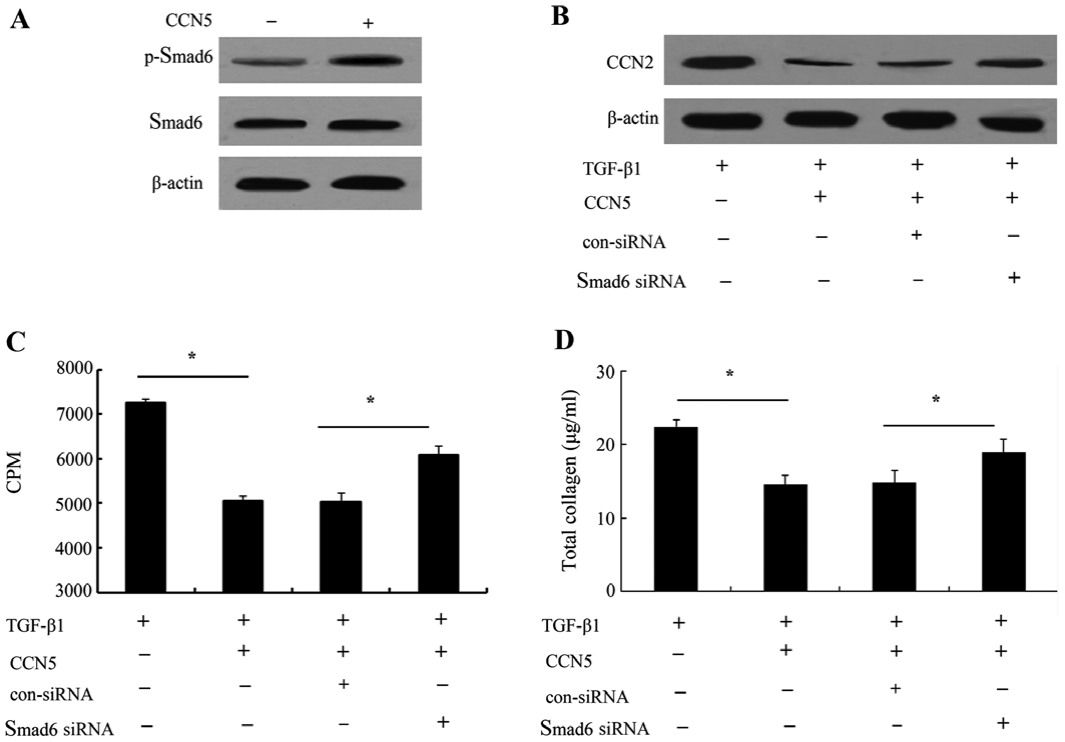
CCN5 functions in TGF-β1-induced proliferation and profibrotic phenotype through the Smad6-CCN2 pathway. To investigate the underlying mechanism, the effect of CCN5 on Smad6 signaling was determined (A). After silencing Smad6 levels by preconditioning with its specific siRNA, the expression levels of CCN2 were measured (B). The function of Smad6 in CCN5-triggered inhibitory effects on fibroblast proliferation (C) was ascertained by [^3^H]-TdR. The corresponding effect on collagen contents was also determined (D). ^*^P<0.05.

To clarify the underlying mechanisms involved in the CCN5-induced anti-fibrotic function, we investigated Smad6 signaling due to the critical role it plays in blocking TGF-β1-triggered other Smad signaling (20,21). As shown in Fig. 6A, the elevated CCN5 expression markedly increased the phosphorylation levels of Smad6. When CCN5 expression was upregulated, CCN2 protein levels induced by TGF-β1 were markedly inhibited. However, silencing Smad6 signaling obviously ameliorated this inhibitory effect on CCN2 levels, suggesting that CCN5 was able to abrogate TGF-β1-induced CCN2 expression levels by Smad6 signaling (Fig. 6B). Furthermore, the Smad6 pathway was blocked with its specific siRNA, and cell proliferation was markedly increased in the CCN5 and TGF-β1-treated groups compared with the control group (Fig. 6C). Of note, the inhibitory function of CCN5 on collagen production was notably decreased (Fig. 6D). These results suggested that the Smad6-CCN2 pathway was involved in the anti-fibrotic function of CCN5.

## Discussion

Postoperative epidural fibrosis, the development of dense and thick scar tissue adjacent to the dura mater after lumbar laminectomy and discectomy, causes compression and stretching of the nerve root and results in persistent back and leg pain (22,23). Although multifactorial factors are involved in the process of failed spine surgery, epidural fibrosis is ranked as the critical contributor to unfavorable clinical outcomes and recurring symptoms (24,25). Given the pivotal roles of scar formation in epidural fibrosis, the prevention of scar formation has received more attention in order to improve spine surgery. In this study, we constructed the post-laminectomy rat models and found an obvious downregulation of CCN5 in scar tissues, suggesting a potentially important role of CCN5 during the development of scar formation and subsequent epidural fibrosis.

Fibrosis often occurs in response to wound repair by excessive scar formation following stimulation such as surgery (1). During this process, fibroblasts are believed to be the prominent participators (3,5). Their proliferation and migration into the wound may contribute to wound repair. Scar is considered to be created by fibroblast proliferation, when inhibiting proliferation, the degree of epidural adhesion was significantly attenuated after laminectomy (3). The TGF-β superfamily is known to exert a number of functions in various physiological processes, including embryonic development, wound repair and cell proliferation (16,26). TGF-β1 ranks as a crucial profibrotic protein and is associated with fibroblast chemotaxis, prolife ration, and ECM deposition (27,28). In this study, we successfully constructed CCN5-ovexpressed cell models. Further analysis revealed that CCN5 upregulation in fibroblasts abrogated cell viability in response to TGF-β1. Consistent with these results, TGF-β1-elevated cell proliferation was significantly attenuated, indicating an important role of CCN5 in fibroblast cell survival and proliferation.

The fibroblasts involved in scarring and contraction are myofibroblasts, which are specialized contractile fibroblasts. It is widely accepted that fibroblasts can convert into profibrotic myofibroblasts following stimulation, which are proven to be increased within fibrotic lesions and contribute to excessive scar formation (29). TGF-β1 is a central regulator for fibrotic response. Its stimulation can transform fibroblasts into myofibroblasts and promotes the synthesis and deposition of ECM, which plays an important role in wound repair and scar formation (28). To clarify the effect of CCN5 during scar development, we analyzed CCN5 function in the anti-fibrotic process. In this study, TGF-β1 triggered an increase in α-SMA and collagen type I expression. Following transfection with LV-CCN5, the upregulation of α-SMA, a specific marker of myofibroblasts, was markedly decreased. Furthermore, CCN5 overexpression reduced the COL1A1 expression levels, which induced collagen I formation, the most abundant product of fibrosis (30). Of note, CCN5 upregulation obviously attenuated TGF-β1-induced total collagen content. Therefore, the results confirm a potential anti-fibrotic effect of CCN5 during the development of EF.

CCN2 is known to be an important profibrotic mediator and is overexpressed in various fibrotic diseases. It often acts as a downstream effector of TGF-β signaling to promote scar tissue production, while suppressing its expression prevents a progressive fibrotic response to TGF-β (11). Furthermore, blocking CCN2 levels with its specific antisense oligonucleotides significantly downregulate myofibroblast numbers and collagen formation, and limits scar hypertrophy (10). Previous findings have shown an opposing role of CCN5 and CCN2 during cardiac fibrosis (14). In this study, we observed an obvious upregulation of CCN2 and downregulation of CCN5 in scar tissues following laminectomy. Further analysis confirmed that CCN5 overexpression markedly decreased CCN2 expression levels induced by TGF-β1 stimulation, indicating a potential anti-fibrosis role of CCN5 by CCN2 signaling.

TGF-β1 is considered a key mediator in fibrosis progression by activating its downstream Smad signaling pathway. The Smad family has been confirmed to be involved in various fibrotic diseases (31-33). Unlike other Smad members, Smad6 can prevent the phosphorylation of Smad members and then act as a negative feedback regulator of the TGF-β superfamily-mediated pathway (21). Previous findings have shown that blocking CCN5 expression increases Smad6 levels (34). Thus, to explore the underlying mechanism involved in the CCN5-induced anti-fibrotic role, Smad6 signaling was analyzed. The results showed that CCN5 upregulation enhanced the phosphorylation of Smad6. When blocking Smad6 with its specific siRNA, the inhibitory effect of CCN5 on CCN2 levels induced by TGF-β1 was notably ameliorated. Smad6 siRNA transfection obviously restored CCN5-inhibited cell proliferation and collagen contents.

In conclusion, this study demonstrated the marked downregulation in CCN5 levels in scar tissues after laminectomy. In this study, CCN5 overexpression significantly abrogated fibroblast proliferation, viability and the profibrotic phenotype of fibroblasts through Smad6-CCN2 signaling, which may contribute to scar formation. Therefore, our findings provide prominent insight into the degree to which CCN5 exerts a potential anti-fibrotic effect during the development of epidural fibrosis. Future studies are required to prove the effect and precise mechanisms of action of CCN5 in ameliorating epidural fibrosis following laminectomy *in vivo*.
